# Character of the Fevers *Prevalent at Chicago, Illinois, in* 1838, ’39

**Published:** 1839-11-09

**Authors:** Daniel Brainard

**Affiliations:** Chicago, Ill.


### CHARACTER OF THE FEVERS *prevalent at Chicago*, *Illinois*, *in* 1838, '39. By Daniel Brainard, M. D.

To the Editors of the Medical Examiner.

Gentlemen,—In accordance with a suggestion in a late number of your journal, I send you a short account of the fevers which prevailed at Chicago during the present and past years.

The southwestern shore of Lake Michigan is low, and the country for several miles adjoining is level; but it rises gradually into "rolling prairies," which are covered with tall grass, and studded with island groves. Between this clear lake, on the one hand, and these fertile prairies on the other, the new city of Chicago is built. The dwellings are principally of wood, and many of them are of the poorer kind. The inhabitants are a collection from nearly every quarter of the globe, although the construction of the Illinois and Michigan canal has, of late, given the preponderance to those from the "Emerald Isle." The climate in summer and autumn, when southern and western winds prevail, is extremely mild; but in the winter, when the winds are from the north, it is cold and bleak.

During the months of July and August, 1838, the remittent fever made its appearance. It usually commenced by languor, loss of appetite, and diarrhœa. These were followed by pains in the head and limbs, chills or rigours, frequent pulse, burning heat, and great thirst. In some cases, instead of diarrhœa, there was constipation. The tongue was in some cases dry and brown, in others brown, but moist, and covered with a thick coat, in others still it was red and denuded. The skin was usually hot and dry during the whole course of the disease, sometimes the extremities were cold, and in a few instances the skin was moist during the remission. Nausea and vomiting were common attendants of the first stage. Tenderness on pressure of the epigastrium occurred in about half the cases. The urine was usually scanty and red, until convalescence.

The duration of the disease varied from three or four days to as many weeks. Most frequently, after the slight premonitory symptoms mentioned above, a slight chill occurred, which was followed for twelve or twenty-four hours with moderate heat, thirst, &c., when, without any recurrence of chill or heat, the patient was restored to health. In cases a little more severe the chill and heat recurred a second and third time, prolonging the disease to six or eight days. In still severer cases the rigor was severe and protracted, followed by intense heat and pain. In the worst cases the hot did not succeed to the cold stage, but the patient had cold extremities, and was not sensible of pain.

The subjects of this fever are mostly the labourers on the Illinois and Michigan canal, and the settlers along the larger streams. In the immediate vicinity of the lake, on the west side, but few cases occur. The traveller, Dwight, (as quoted in Armstrong's Lectures,) observes that the borders of clear lakes are healthy, while those of muddy and stagnant waters are the reverse. This observation I believe to be generally correct. In regard to the proportion of cases that prove fatal, it is difficult to speak with certainty; but, according to my observations, about one in sixteen of the severer cases terminates fatally. Some of these are in very unfavourable subjects.

The treatment varies with each case. In the mildest, gr. viij. of blue pill mass, given at evening, followed in the morning by ℥j. castor oil, with confinement to bed, were sufficient to effect a cure. In severer cases, calomel, followed by castor oil, emetics, general blood-letting, if the patient was of good habit, or if there were marks of severe local affection, were employed in the first stages; in the later periods, local bleeding, blisters, and calomel, in smaller portions, were useful. Cold sponging, or effusion, with iced water for drink, was of the greatest use, when the febrile action was high. The usual routine of treatment in northern Illinois, is calomel, in large doses, from first to last. Forty grains is considered a moderate dose, and ʒjss. is often given. Much harm, I am sure, results from the indiscriminate use of this article. In persons of delicate constitution, and in those weakened by previous disease, it is apt to produce ptyalism, and in all cases it is liable to leave behind chronic irritation of the bowels, which is not free from danger.

In August, 1839, the fever occurred, commencing much in the same manner as in '38. It ceased early in September. It differed from that of 1838, in being often accompanied by severer local affections, somnolence, and profuse sweats. General blood-letting was oftener required, but had to be used in the first stage, and in moderation, or it was injurious. Tonics, wine, bark, sul. quiniæ with wine, were useful in the later stages of nearly every case. In the fevers of '38, they were but rarely required. The disease of this season was less fatal than in the previous year. Out of twenty-eight cases I lost none.

I witnessed the examination of two persons dead of this fever. The first died on the sixth day of the disease, during the operation of a lobelia emetic. The mucous membrane of the stomach and duodenum were red, thickened and softened in several spots. The mucous coat of the ileum was red and thickened, and covered with a dark mucous. The elliptical patches of the ileum were thickened, and of a light color; the mesenteric glands, opposite to these thickened patches, were redder than the rest. The mucous coat of the colon and rectum was, in spots, thickened, softened, and covered with green mucous.

The liver was of its natural size and colour, but on being cut or torn, its vessels poured out blood and bile freely. The spleen, pancreas, and kidneys were healthy.

The second subject died on the twenty-first day of the disease. He had been convalescent, but had a relapse, with diarrhœa, for which he took several large doses of calomel; the diarrhœa increased, and, after copious discharges of blood, by stool, he died.

The mucous membrane of the stomach was red, and thickened in some small spots along the greater curvature; that of the duodenum and upper portion of the ileum was but slightly reddened and softened in spots. In the lower yard of the ileum it was throughout of a dark brown colour, thickened and softened. The intestine at this part was contracted in size, and filled with mucous, tinged with blood. The same appearance, in a smaller degree, extended through the colon and rectum; in the latter were some small ulcerations.

The liver was of the natural size but pale; in the other abdominal viscera no marks of disease were observed.

I was unable to obtain full histories of these cases, or to make further examinations of the bodies.

The above is but a slight outline of the more prominent features of this disease, but I hope it is sufficient to enable the experienced practitioner to discover somewhat of its character and treatment. Of the agues and other diseases of the country I have purposely omitted to speak at present.

*Chicago, Ill., October*, 1839.

